# Non-ischemic Cerebral Enhancing (NICE) Lesions Following Endovascular Treatment of Intracranial Aneurysms: A Case Report

**DOI:** 10.7759/cureus.80667

**Published:** 2025-03-16

**Authors:** Christos Tzerefos, Ioannis Ioannidis, Georgios P Karagiorgas, Mariana Vlychou, Kostas N Fountas

**Affiliations:** 1 Department of Neurosurgery, University Hospital of Larissa, Faculty of Medicine, University of Thessaly, Larissa, GRC; 2 Department of Radiology, University Hospital of Larissa, Faculty of Medicine, University of Thessaly, Larissa, GRC

**Keywords:** endovascular therapy (evt), flow diverter, intracranial aneurysms, non-ischemic cerebral enhancing (nice) lesions, stent-assisted coiling

## Abstract

Non-ischemic cerebral enhancing (NICE) lesions are a rare complication following endovascular therapy (EVT) for intracranial aneurysms, presenting as delayed-onset enhancing lesions on MRI. While their pathophysiology remains unclear, NICE lesions can pose diagnostic challenges due to their resemblance to neoplastic or infectious processes. We report a case of a 67-year-old female with incidental anterior communicating artery (ACom) and posterior inferior cerebellar artery (PICA) aneurysms treated with EVT using a flow diverter for the PICA aneurysm and stent-assisted coiling for the ACom aneurysm. Three months post procedure, a follow-up MRI revealed punctate, nodular, and annular enhancing lesions with peri-lesional edema, consistent with NICE lesions. Despite these findings, the patient remained asymptomatic, reporting only subjective fatigue. She was treated with a one-month course of glucocorticosteroids, leading to symptom resolution. Serial MRI over one year demonstrated a reduction in edema, though new lesions appeared. This case underscores the importance of long-term imaging surveillance following EVT for cerebral aneurysms, as NICE lesions may persist, regress, or evolve over time. Although the clinical course may be benign in some patients, the presence of persistent or newly emerging lesions raises concerns regarding their underlying mechanisms and potential long-term impact. Further research is needed to better understand the pathophysiology, optimize management strategies, and refine follow-up protocols for patients with NICE lesions after EVT.

## Introduction

Non-ischemic cerebral enhancing (NICE) lesions are a rare complication following endovascular therapy (EVT) for intracranial aneurysms, with reported incidence rates ranging from 0.05% to 1% [1-3]. These lesions typically present as delayed-onset punctate, nodular, or annular MRI enhancements with peri-lesional edema, often localized within the vascular territory of the treated aneurysm [3].

Although the precise pathophysiological mechanisms remain ambiguous, NICE lesions are postulated to arise from foreign body emboli instead of hypersensitivity responses to EVT devices [4]. Clinically, these lesions may present with a spectrum of symptoms ranging from asymptomatic cases to severe neurological impairments, encompassing headaches, focal neurological deficits, and seizures [1,2]. Despite the generally benign nature of their clinical progression, the presence of persistent MRI enhancement may obscure the differentiation from neoplastic or infectious lesions, underscoring the necessity of careful clinical correlation, follow-up imaging, and advanced imaging techniques to accurately distinguish NICE lesions from other enhancing brain pathologies.

Here, we present a case of a 67-year-old female who underwent coiling and stenting for two brain aneurysms and subsequently developed asymptomatic non-ischemic enhancing lesions on her follow-up MRI scan.

## Case presentation

**Figure 1:**
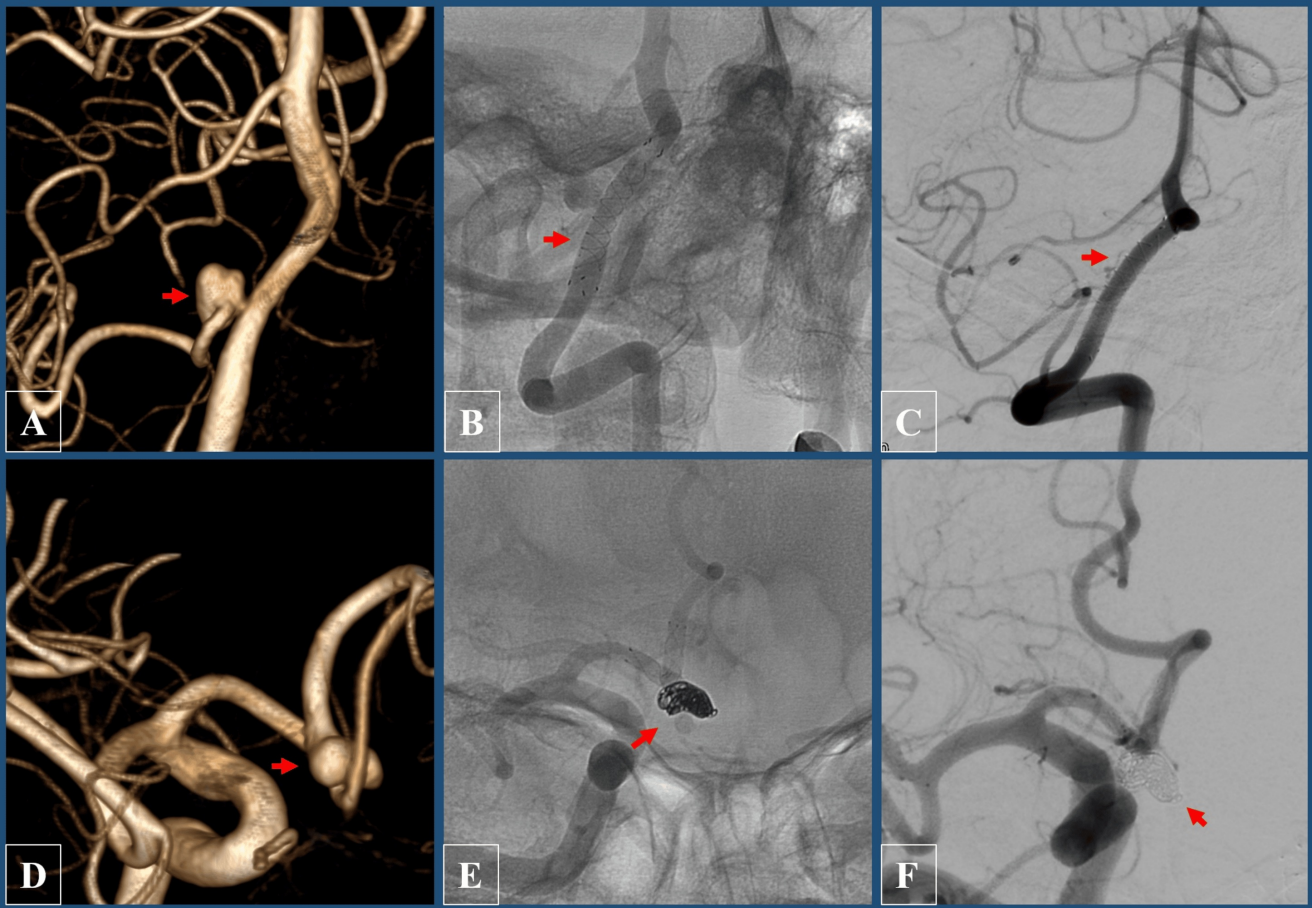
Digital subtraction angiography (DSA) images demonstrating intraoperative and postoperative outcomes. Upper row: Posterior inferior cerebellar artery (PICA) aneurysm. (A) Preoperative 3D rotational DSA showing the aneurysm (red arrow). (B) Immediate intraoperative outcome following treatment with a flow diverter (red arrow). (C) One-year follow-up DSA showing no recurrence or residual aneurysm (red arrow). Lower row: Anterior communicating (ACom) artery aneurysm. (D) Preoperative 3D rotational DSA showing the aneurysm (red arrow). (E) Immediate intraoperative outcome following coil embolization (red arrow). (F) One-year follow-up DSA showing no recurrence or residual aneurysm (red arrow).

A 67-year-old female, previously asymptomatic, was diagnosed with two intracranial aneurysms: an anterior communicating (ACom) artery aneurysm and a posterior inferior cerebellar artery (PICA) aneurysm arising from the right vertebral artery (Figures 1A, 1D). The aneurysms were incidentally detected on an MRI scan performed for headaches. After a thorough discussion with the patient, an endovascular approach was chosen.

On November 14, 2023, the patient underwent successful endovascular treatment for both aneurysms under general anesthesia after preparation with dual antiplatelet therapy (DAPT) consisting of acetylsalicylic acid 100 mg and ticagrelor 90 mg twice daily. The PICA aneurysm was treated with stent placement using a flow diverter (FRED X, Microvention Terumo, Aliso Viejo, CA). In contrast, the ACom aneurysm was managed with stent-assisted coiling (LVIS Jr, Microvention Terumo) involving the right A1 and A2 segments (Figures 1B, 1E). The procedure was completed without complications, and the patient recovered well postoperatively. She remained neurologically intact and was discharged in stable condition two days later.

**Figure 2:**
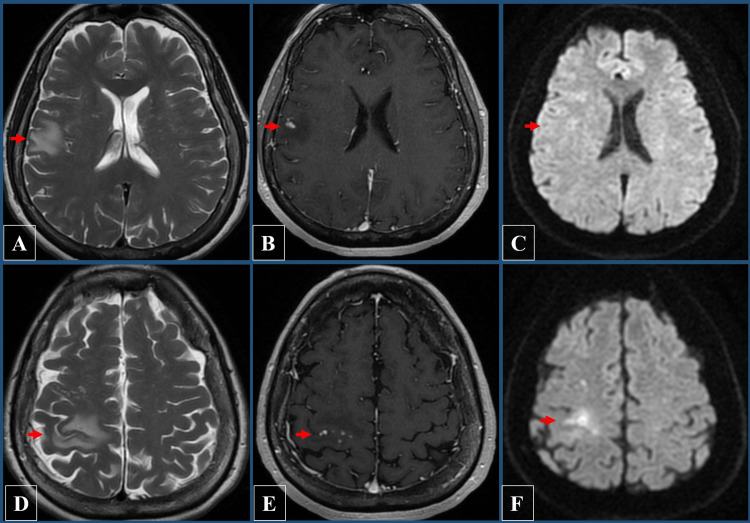
Magnetic resonance imaging (MRI) demonstrating axial images at three-month follow-up. (A, D) T2 fluid-attenuated inversion recovery (FLAIR) images showing lesions with peri-lesional edema in the right hemisphere (red arrows). (B, E) T1-weighted post-contrast images demonstrating annular enhancing lesions, consistent with non-ischemic cerebral enhancing (NICE) lesions (red arrows). (C, F) Diffusion-weighted imaging (DWI) showing the lesions with restricted diffusion (red arrows).

At her three-month follow-up, an MRI revealed a delayed-onset punctate, nodular, and annular enhancing lesion with peri-lesional edema in the right hemisphere, consistent with NICE lesions (Figure 2). Despite these findings, the patient remained clinically asymptomatic, with no focal neurological deficits, but reported subjective fatigue. She was started on a one-month course of glucocorticosteroids, which led to an improvement in her subjective symptoms. Following this episode, she did not require further corticosteroid treatment.

**Figure 3:**
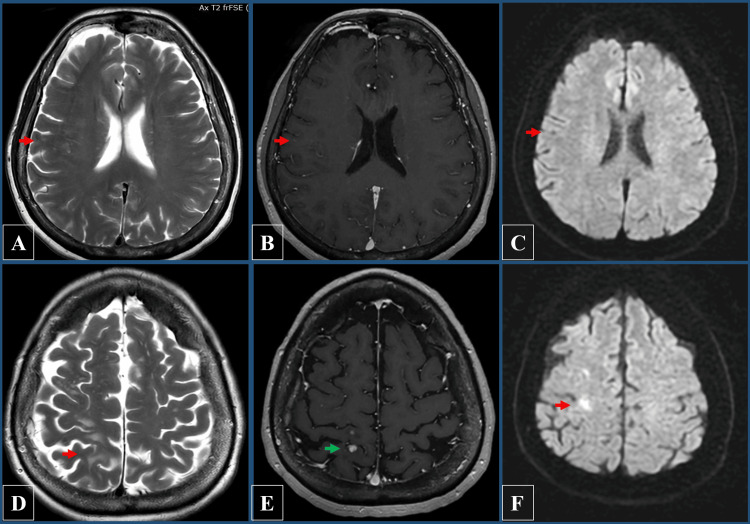
Magnetic resonance imaging (MRI) demonstrating axial images at one-year follow-up. (A, D) T2 fluid-attenuated inversion recovery (FLAIR) images showing a reduction in peri-lesional edema (red arrows). (B, F) T1-weighted post-contrast images showing a decrease in the number of nodular enhancing lesions (red arrow), while new lesions emerged with the most prominent being 4 mm in diameter (green arrow). (C, F) Diffusion-weighted imaging (DWI) showing the lesions with restricted diffusion (red arrows).

She was closely monitored with repeated MRI scans, and over the course of 18 months, serial imaging demonstrated stability of the lesion, with an apparent reduction in edema. While the number of nodular enhancing lesions decreased, interestingly, new enhancing lesions appeared (Figure 3). While most of the remaining lesions were located within the white matter and cortical regions, we did not identify any perivascular lesions in the vicinity of the major vessels of the circle of Willis. A follow-up digital subtraction angiography (DSA) performed one year later confirmed no recurrence or residual aneurysm (Figures 1C, 1F). The patient remained asymptomatic throughout the follow-up period, with no recurrence of her subjective symptoms.

## Discussion

NICE lesions are rare complications following EVT for cerebral aneurysms, characterized by delayed-onset punctate, nodular, or annular MRI enhancements with peri-lesional edema. The reported incidence of NICE lesions varies between studies, ranging from 0.5% to 1%, depending on the population studied and the follow-up protocol employed [1-3]. In one significant study by Richter and colleagues involving 1201 patients treated with EVT, NICE lesions were observed in 1% of cases (12 out of 1201), highlighting the rarity of this condition even in large cohorts [2].

Presentation

The clinical presentation of NICE lesions varies widely, ranging from asymptomatic cases to severe symptoms, such as hemiplegia and cognitive impairment [2]. In symptomatic cases, the location of the lesion often determines the specific clinical manifestations. According to a study by Richter et al., headache is the most reported symptom leading to the diagnosis of NICE lesions, observed in 33.3% of cases. Other symptoms reported in the same study include epileptic seizures (13.3%), cognitive impairment (13.3%), and focal neurological deficits (20.0%). These findings are consistent with observations from other studies [2,3,5,6]. Some patients may initially present as asymptomatic and later develop symptoms [6].

Symptoms can appear rapidly and progress quickly [7]. On average, they manifest approximately 15 days after EVT, with a reported range of two to 40 days. However, late presentations have also been documented. For instance, Guetarni et al. described a case of a patient who developed seizures and subcortical edema affecting multiple regions of the right hemisphere 17 months after EVT [6].

In contrast, our patient exhibited a completely asymptomatic clinical course despite notable MRI findings, including delayed-onset punctate and nodular enhancing lesions with peri-lesional edema. Moreover, although imaging revealed not only persistence but the emergence of new lesions, our patient remained neurologically stable throughout an extended 18-month follow-up, highlighting an atypically benign clinical progression compared to previously documented cases. It is noted that NICE lesions may not always necessitate aggressive clinical intervention. Instead, conservative monitoring may be appropriate in asymptomatic patients, suggesting that clinical decision-making should be individualized and guided by clinical presentation rather than imaging alone.

Imaging

MRI is the gold standard modality for detecting NICE lesions following EVT for cerebral aneurysms [5]. NICE lesions typically present as delayed-onset punctate, nodular, or annular enhancing foci with associated peri-lesional vasogenic edema distributed in the vascular territory of the treated aneurysm [2,4,6]. The lesions frequently spare the overlying cortex and are primarily located in the subcortical white matter or watershed zones [2,7]. The initial lesion burden observed on MRI varies, with a median of 36 lesions reported in one study [6].

Key MRI sequences provide critical insights into the nature of these lesions. On T2-weighted and fluid-attenuated inversion recovery (FLAIR) sequences, the lesions appear hyperintense due to surrounding edema [3,7]. Some lesions exhibit hypointense signals on gradient recalled echo (GRE) imaging, indicating potential microhemorrhages [7]. Diffusion-weighted imaging (DWI) may show hyperintense foci with corresponding lowered apparent diffusion coefficient (ADC) values, although findings are often complicated by imaging artifacts such as Nyquist ghosting [3,7]. Persistent enhancement on contrast-enhanced MRI is observed in up to 71% of cases, lasting for a median duration of 13 weeks [6].

Leptomeningeal and cortical enhancements have also been reported, with enhancements demonstrating a smooth, thin ring pattern in some cases. Follow-up MRI studies commonly show significant regression of the enhancing lesions and associated edema, although late manifestations have been described, such as subcortical edema and seizures occurring up to 17 months post EVT [6,7].

These imaging findings highlight the critical role of MRI in diagnosing and monitoring NICE lesions, with sequences such as T2-weighted, GRE, and DWI providing valuable diagnostic information. In our case, serial imaging demonstrated the stability of the lesion, with a significant reduction in edema. However, we had new enhancing lesions that developed over time.

Pathophysiology

The pathophysiology of delayed NICE lesions following EVT for cerebral aneurysms remains unclear, though several theories have been proposed. One hypothesis suggests that these lesions result from foreign body emboli rather than nickel allergy, as skin patch testing has shown no allergic reactions to the endovascular devices used [4]. Granulomatous lesions may arise from foreign body reactions, evidenced by pathological examinations indicating inflammatory responses such as eosinophilic leukocytoclastic vasculitis, which could signify hypersensitivity angiitis associated with embolic foreign material. Another theory posits that shedding hydrophilic coatings from microcatheters may trigger granulomatous foreign-body reactions, leading to punctate lesions observable on neuroimaging. Repeated friction during procedures can damage the inner walls of microcatheters, resulting in conglomerated fragments that may act as downstream emboli, contributing to the development of NICE lesions [8]. The inflammatory response to these dislodged materials can culminate in granulomatous angiitis and the formation of microabscesses, which are typically identified on MRI as punctate, nodular, or annular foci exhibiting enhancement. This inflammatory response may elicit neurological manifestations that vary in severity and can improve with corticosteroid therapy [9]. In our case, the PICA aneurysm was treated with a flow diverter, requiring minimal catheter manipulation, thereby reducing the likelihood of microcatheter-induced friction. In contrast, the ACom artery aneurysm was treated with multiple devices (stent-assisted coiling with the microcatheter jailed within the aneurysmal sac), which could have led to increased friction against the inner walls of the microcatheters. The variability in patient responses underscores the need for further research and long-term follow-up to fully understand the mechanisms behind NICE lesions and their implications for patient outcomes.

Differential diagnosis

When encountering enhancing lesions on post-procedural MRI, it is essential to consider a broad differential diagnosis to accurately distinguish NICE lesions from other pathologies. Ischemic infarcts typically appear early in the post-procedural period and demonstrate restricted diffusion on DWI, unlike NICE lesions, which are non-ischemic and tend to appear later [3,6]. Tumefactive demyelination may exhibit similar imaging characteristics, including contrast enhancement and surrounding edema, but clinical context and supportive imaging findings, particularly on DWI, aid in differentiation [6]. Infections or abscesses, another consideration, usually correlate clinically with fever, elevated inflammatory markers, and systemic symptoms, making laboratory confirmation critical [6]. Lastly, although uncommon, neoplastic lesions such as gliomas or metastases should be considered when new or enlarging enhancing lesions appear post-procedurally, often requiring biopsy for definitive diagnosis [6]. NICE lesions, in contrast, typically remain stable or regress spontaneously over time, highlighting the importance of careful longitudinal imaging follow-up to differentiate them from other pathologies.

## Conclusions

The findings highlight the complexity of managing patients with NICE lesions, emphasizing the importance of tailored treatment approaches and ongoing monitoring to mitigate potential complications.
